# A Novel, Deep Learning-Based, Automatic Photometric Analysis Software for Breast Aesthetic Scoring

**DOI:** 10.1055/a-2190-5781

**Published:** 2024-02-07

**Authors:** Joseph Kyu-hyung Park, Seungchul Baek, Chan Yeong Heo, Jae Hoon Jeong, Yujin Myung

**Affiliations:** 1 Department of Plastic and Reconstructive Surgery, Seoul National University Bundang Hospital, Seoul National University College of Medicine, Seongnamsi, Gyeonggi-do, Republic of Korea

**Keywords:** breast cancer, aesthetics, deep learning

## Abstract

**Background** Breast aesthetics evaluation often relies on subjective assessments, leading to the need for objective, automated tools. We developed the Seoul Breast Esthetic Scoring Tool (S-BEST), a photometric analysis software that utilizes a DenseNet-264 deep learning model to automatically evaluate breast landmarks and asymmetry indices.

**Methods** S-BEST was trained on a dataset of frontal breast photographs annotated with 30 specific landmarks, divided into an 80–20 training–validation split. The software requires the distances of sternal notch to nipple or nipple-to-nipple as input and performs image preprocessing steps, including ratio correction and 8-bit normalization. Breast asymmetry indices and centimeter-based measurements are provided as the output. The accuracy of S-BEST was validated using a paired *t* -test and Bland–Altman plots, comparing its measurements to those obtained from physical examinations of 100 females diagnosed with breast cancer.

**Results** S-BEST demonstrated high accuracy in automatic landmark localization, with most distances showing no statistically significant difference compared with physical measurements. However, the nipple to inframammary fold distance showed a significant bias, with a coefficient of determination ranging from 0.3787 to 0.4234 for the left and right sides, respectively.

**Conclusion** S-BEST provides a fast, reliable, and automated approach for breast aesthetic evaluation based on 2D frontal photographs. While limited by its inability to capture volumetric attributes or multiple viewpoints, it serves as an accessible tool for both clinical and research applications.

## Introduction

Breast aesthetics is important in various medical fields, such as plastic surgery, radiology, and oncology. 1 2 3 4 Evaluations of breast aesthetics have traditionally been subjective and inconsistent, relying on assessments by physicians and patients. Subjective assessments of breast aesthetics are prone to interobserver variability and may not accurately reflect the true characteristics of breast morphology. Moreover, they may not be reproducible over time, leading to inconsistency in follow-up evaluations. As a result, there is an increasing demand for objective and standardized ways of evaluating breast aesthetics that can deliver more reliable and consistent results.

As technology advances, there is a greater interest in developing computer-assisted analysis. Recently, software have been developed to provide quantitative assessments of breast landmarks and features. BCCT.core, Breast Analyzing Tool (BAT), Breast Aesthetic Scale, and other software have been introduced over the past two decades. 5 6 7 However, the need for faster and automated photometric software still exists, since current software require manual landmark and feature selection, which can be time-consuming.

To improve clinical and research efficiencies, we developed the Seoul Breast Esthetic Scoring Tool (S-BEST), a photometric analysis software that aims to provide improved measurement and assessment of breast landmarks and features. S-BEST utilizes deep learning-based models to detect landmarks automatically, provide breast asymmetry indices, and measure distances within a few seconds on modern computers.

## Methods

We developed S-BEST for photometric analysis, which requires a frontal photo of the breast as input. All photographs were taken by our in-hospital photographer with predesigned settings (camera to patient distance of 4 m, F/13–16, 55–60 mm, ISO 200, with ceiling-mounted flash and blue background). The DenseNet-264 model was implemented using Python 3.5 and Tensorflow 1.5, within an Anaconda environment and developed in PyCharm. Our dataset comprised frontal breast photographs annotated with 30 landmark points specific to breast features. The landmarks included the sternum, the umbilicus, the nipples, and breast footprints (13 points for each side). All 30 points were manually marked by a single plastic surgeon to improve the integrity of the training set. The dataset was partitioned in an 80–20 ratio for training and validation, respectively. The model's architecture was designed to be compact, resulting in a model size of approximately 200 MB, making it suitable for deployment in various computational settings. Prior to training, image preprocessing steps were undertaken to ensure consistency across the dataset. These steps included correcting the image ratio, standardizing image sizes, and performing 8-bit normalization to scale pixel values. The training process was configured to optimize the model's ability to accurately identify and measure breast landmarks.

The final program with the deep learning-based model requires at least one of three actual measurements: sternal notch to the right nipple, sternal notch to the left nipple, and nipple-to-nipple distances. If these measurements are unavailable, a reasonable value can be input. However, distance estimations and dimension-based breast asymmetry indices calculated will not be accurate. In such cases, dimensionless indices can still be used accurately.

**Fig. 1:**
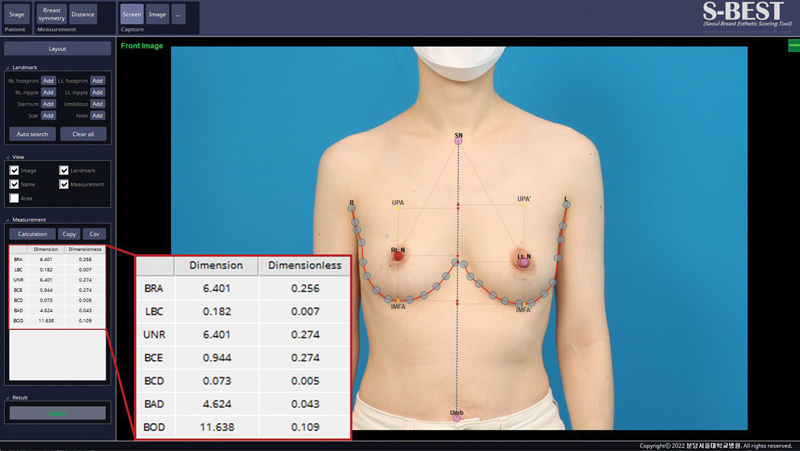
Screenshot capture of the S-BEST output page. Landmarks are automatically detected based on deep learning models. Breast asymmetry indices are given on the bottom left. Manual distance measuring can also be performed. BAD, breast area difference; BCD, breast contour difference; BCE, breast compliance evaluation; BOD, breast overlap difference; BRA, breast retraction assessment; LBC, lower breast contour; S-BEST, Seoul Breast Esthetic Scoring Tool; UNR, upward nipple retraction.

**Fig. 2:**
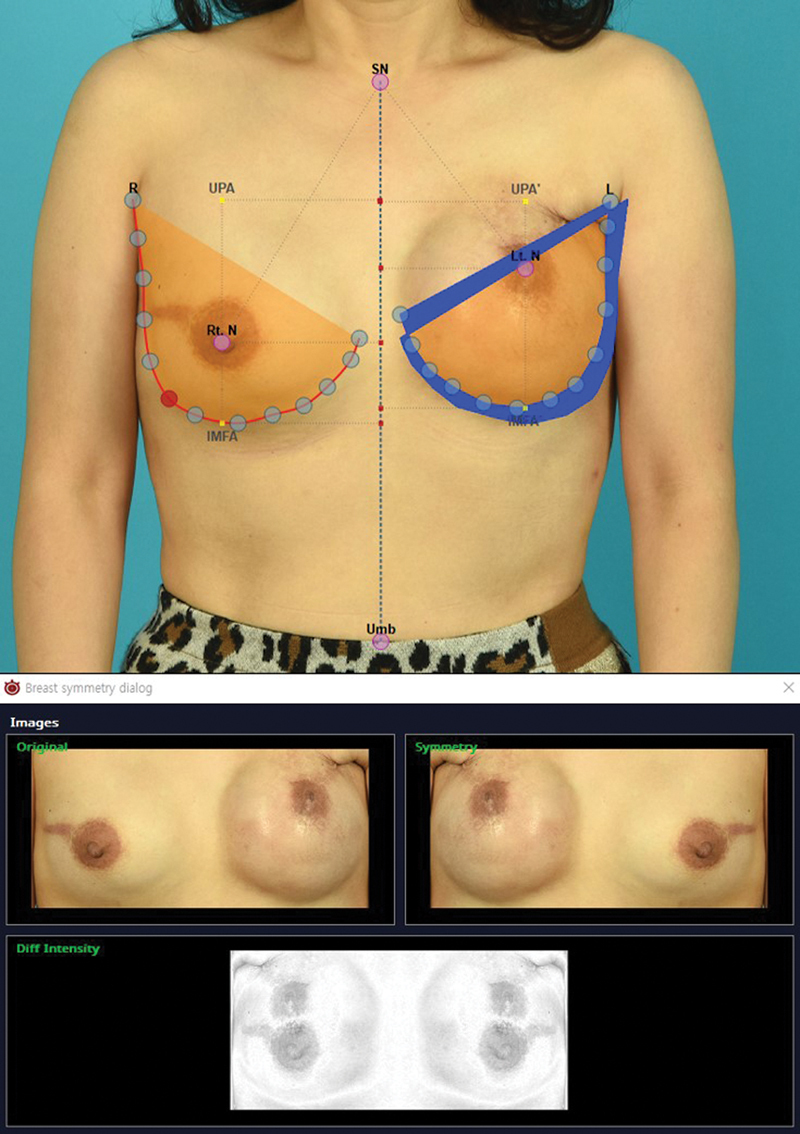
Screenshot capture of the breast symmetry function of S-BEST. (Above) The orange shade indicates the breast footprint, while the blue shade indicates the breast overlap difference (BOD). (Below) An overlap image of the breasts is provided to better visualize the discrepancy in the nipple–areola complex and the footprint between the two breasts. S-BEST, Seoul Breast Esthetic Scoring Tool.

S-BEST outputs automatic landmark localization, breast asymmetry indices calculations 8 (dimension-based and dimensionless), breast symmetry comparison, and a manual distance measurements tool that estimates distance in centimeters ( Fig. 1 ). Breast asymmetry indices provided by the program include breast retraction assessment (BRA), lower breast contour (LBC), upward nipple retraction, breast compliance evaluation, breast contour difference, breast area difference, and breast overlap difference (BOD; Fig. 2 ). 3 8 9 10

**Fig. 3:**
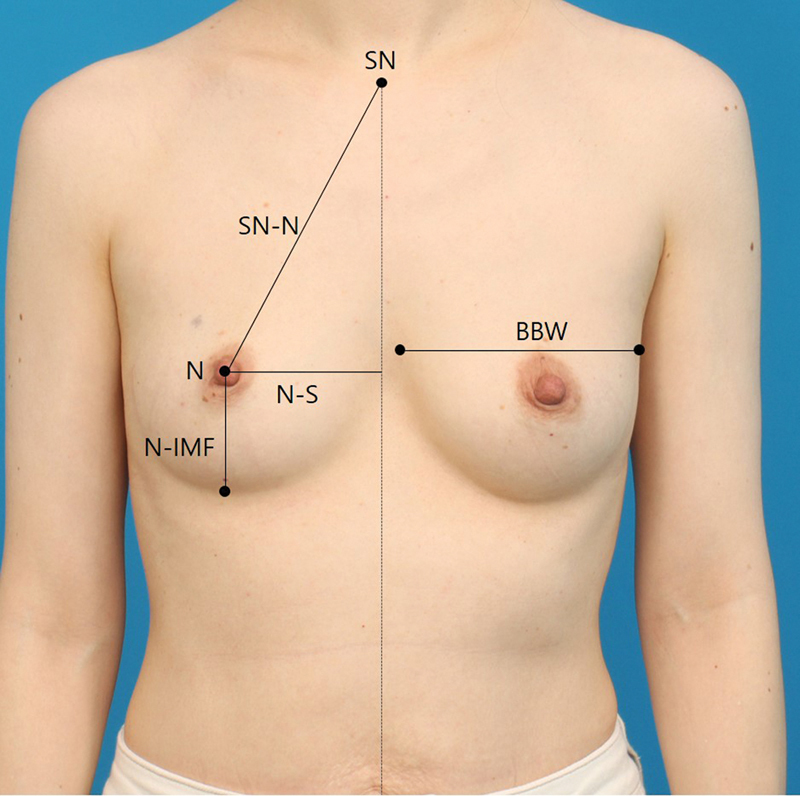
Landmarks and distances used for measurement validation (SN, sternal notch, N, nipple; SN-N, sternal notch to nipple; N-S, nipple to sternum; N-IMF, nipple to inframammary fold; BBW, breast base width).

Since the breast symmetry indices are either centimeter (dimension-based) or pixel (dimensionless)-based calculations, it is necessary to validate S-BEST's accuracy compared with physical measurements. One hundred females diagnosed with breast cancer were included in this validation study. One consecutive patients' photographs taken in January and February of 2023 (to avoid overlap with training datasets) were used. A single plastic surgeon (Y.M.) marked and measured landmarks during the physical examination. The landmarks included the sternal notch, the sternum, the nipples, the inframammary fold, and the umbilicus. The distances measured included sternal notch to nipple, nipple to sternum, nipple to inframammary fold, and breast base width ( Fig. 3 ).

We also used S-BEST to obtain automatic landmark localization and distance measurements. Another plastic surgeon not involved in the initial physical examination (J.K-h.P.) was given frontal photographs and the sternal notch to the right nipple distance to be input into the program. We compared the results of S-BEST and physical measurements (the ground truth) using a paired *t* -test with a 0.05 significance level and Bland–Altman plots. The study was performed in accordance with the principles of the Declaration of Helsinki. The study was approved by the Institutional Review Board of Seoul National University Bundang Hospital (IRB#B-2207-770-101).

## Results

A total of 100 females diagnosed with breast cancer participated in this study, with a mean age of 48.2 years (ranging from 28 to 71 years). The mastectomy specimen weights averaged 189.4 g (28–736 g). The paired *t* -test showed that only the nipple to inframammary fold distance showed statistical significance, while the other measurements showed no statistical significance between physical examination and S-BEST's automatic measurements ( Table 1 ).

**Table 1:** Mean values and paired *t* -test between actual measurements and Seoul Breast Esthetic Scoring Tool's calculations

	Mean Actual (cm)	Mean S-BEST (cm)	Paired *t* -test ( *p* -value)	Mean difference (cm)	Upper limit of agreement (cm) b	Lower limit of agreement (cm) b
Sternal notch to nipple (Rt.)	20.8	21.0	0.47	−0.2	0.7	−1.1
Sternal notch to nipple (Lt.)	20.8	20.8	0.32	0.1	1.4	−1.2
Nipple to sternum (Rt.)	8.5	8.6	0.28	−0.1	1.8	−2.0
Nipple to sternum (Lt.)	8.8	8.8	0.49	−0.1	2.0	−2.2
Nipple to inframammary fold (Rt.)	6.6	4.0	<0.001 a	2.7	7.2	−1.9
Nipple to inframammary fold (Lt.)	6.5	4.0	<0.001 a	2.6	7.0	−1.8
Breast base width (Rt.)	12.6	12.5	0.45	0.1	2.6	−2.4
Breast base width (Lt.)	12.6	12.3	0.46	0.2	2.7	−2.2

Abbreviations: Lt., left; Rt., right; S-BEST, Seoul Breast Esthetic Scoring Tool.

a Statistically significant ( *p-* value < 0.05).

b Mean ± 1.96 × SD.

**Fig. 4:**
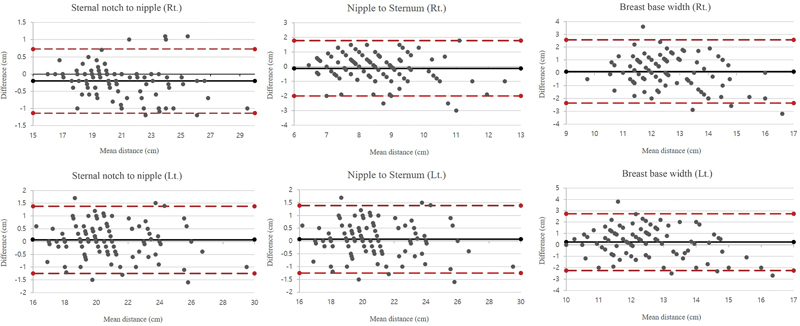
The Bland–Altman plots for the sternal notch to the nipple, nipple to the sternum, and breast base width showed a bias of less than 0.2 cm, with upper and lower limits of agreement of less than 3 cm. Lt., left; Rt., right.

**Fig. 5:**
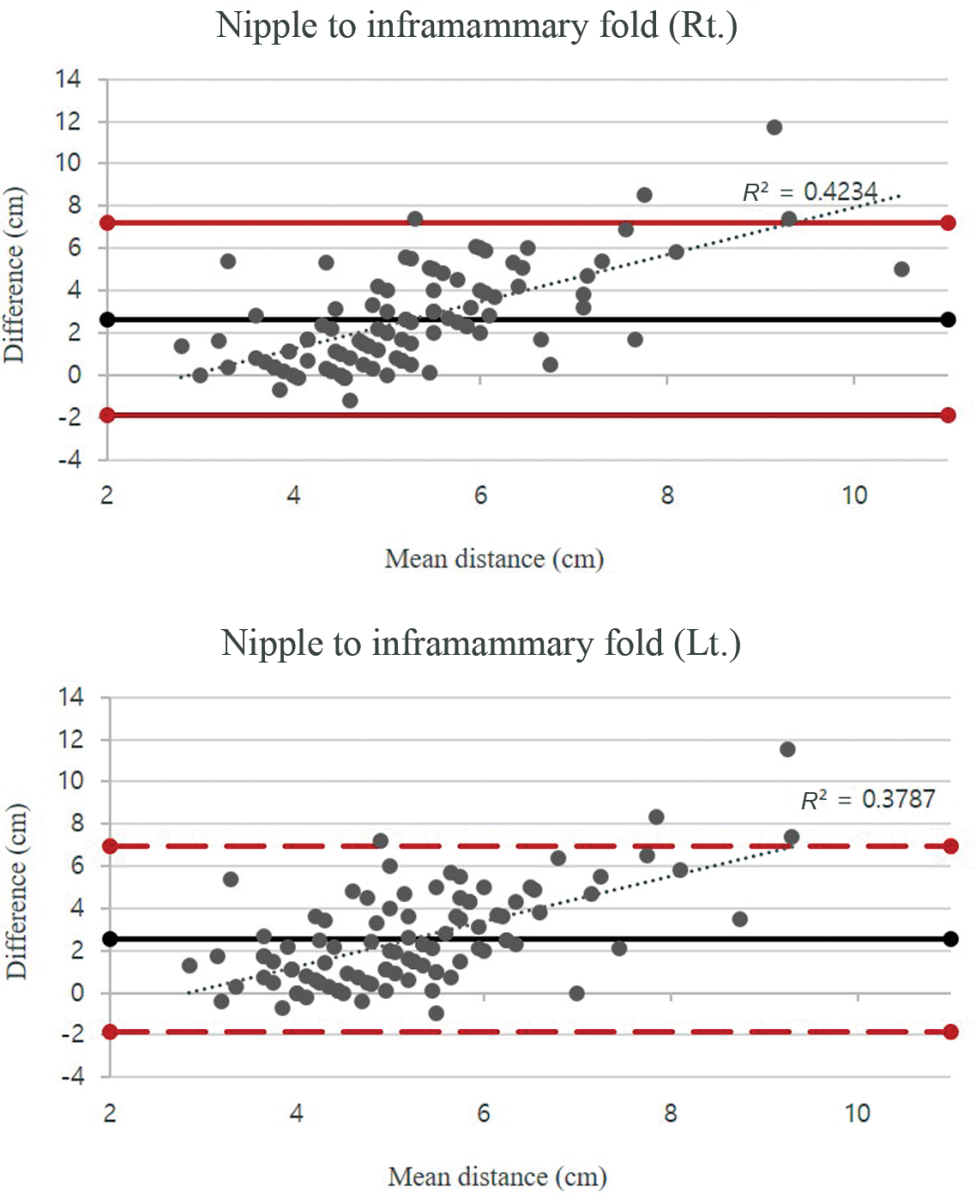
The Bland–Altman plots for the nipple to inframammary fold distance showed a high bias of 2.7 cm, while the upper and lower limits of agreement were 7.2 and −1.9 cm, respectively. The discrepancy between the ground truth and S-BEST measurement rose as the mean nipple to inframammary fold distance increased, resulting in coefficients of determination of 0.4234 and 0.3787 for the right and left sides, respectively. Lt., left; Rt., right.; S-BEST, Seoul Breast Esthetic Scoring Tool.

The sternal notch to nipple, nipple to sternum, and breast base width Bland–Altman plots revealed a bias of less than 0.2 cm, with upper and lower limits of agreement of less than 3 cm ( Fig. 4 ). The nipple to inframammary fold distance, on the other hand, had a significant bias of 2.7 cm, with upper and lower limits of agreement of 7.2 and −1.9 cm, respectively ( Fig. 5 ). The discrepancy between the ground truth and S-BEST measurement rose as the mean nipple to inframammary fold distance increased, resulting in coefficients of determination of 0.4234 and 0.3787 for the right and left sides, respectively.

## Discussion

Patients and health care providers have been looking for new strategies to improve patient satisfaction and quality of life as the survival rate after breast cancer diagnosis has grown over the last several decades. 11 12 13 Maintaining good breast cosmesis after breast cancer operation is one such aspect. To objectively evaluate aesthetic outcomes after breast-conserving surgery, mastectomies, chemotherapy, and radiation therapy, algorithm-based programs are needed. 5 14 15 Several programs have been introduced with great success in providing accurate results. 5 6 7 16 We have noticed the impracticality of using these programs in a clinical setting, especially during outpatient clinic follow-up evaluations, due to the slow processing of the programs caused by the need for manual inputs.

We have developed S-BEST to automatically detect landmarks and provide breast measurements based on a frontal photograph using deep learning to reduce the time needed to evaluate a patient using such a program. Our analysis of the measurements provided by S-BEST compared with the ground truth (physical measurements) showed excellent results. The automatic photometric analysis by S-BEST was accurate for the sternal notch to the nipple, nipple to the sternum, and breast base widths. However, the nipple to inframammary fold distance showed a high bias, with a positive coefficient of determination. This is expected because a more ptotic breast means that the inframammary fold is not clearly visible, and the photometric analysis only measures the distance shown on the photo, while the physical measurement measures the entire length of the lower pole.

Breast symmetry indices, such as BRA, LBC, BAD, and BOD, are essential for evaluating breast reconstruction surgery's success. These indices are based on photometric analysis measurements using a frontal breast image. As our analysis shows, S-BEST provides accurate measurements of breast features used for breast symmetry indices, therefore, these indices are also accurate.

Several other programs are available to provide photometric analysis of breast symmetry and asymmetry. For example, the BAT, Breast Idea, and BCCT.core are commercial programs that automatically measure breast size and asymmetry. 5 6 7 However, these programs still require manual selection of landmarks and do not provide automatic localization of landmarks like S-BEST.

Compared with these programs, S-BEST offers several advantages. First, it provides automatic landmark localization, which saves time and reduces the risk of errors associated with manual landmark selection. This is important, especially during outpatient consultation, which is constrained by time. Second, it offers dimension-based and dimensionless breast asymmetry indices, which can provide more accurate and standardized measures of breast asymmetry. Also, when physical measurements are unavailable, dimensionless indices can be helpful. For example, during a retrospective analysis where actual measurements are missing, aesthetic comparisons between patients can be done using the dimensionless indices. Third, it can be used for both clinical and research purposes, providing valuable information for patient counseling, surgical planning, and outcome evaluation.

S-BEST is a helpful instrument for both clinical and research reasons since it can perform precise and quick photometric measurements. Its automatic landmark localization and extensive measuring possibilities make it a great tool for clinical and research applications. However, the program has some limitations, including reliance on the frontal photo, which can affect measurement accuracy. More research is needed to validate S-BEST's accuracy in a larger patient population and to look into its applicability to other breast conditions like ptosis, breast augmentation, and breast reduction. Future research could also look into how other physical attributes like chest size, height, and body mass index interact with the breast asymmetry indices to provide a more holistic understanding of breast aesthetics.

While the S-BEST tool offers significant advantages in speed and automation for 2D photographic analysis, it is important to recognize the inherent limitations of 2D data in capturing volumetric attributes of the breast. Recent advancements in 3D analysis have indeed provided more reliable metrics for assessing breast aesthetics. However, 3D analysis often requires specialized equipment and software, potentially limiting its accessibility and widespread adoption. Also, most software cannot render 3D analysis based on 2D photographs, limiting their use on older data without 3D scanning. S-BEST aims to provide a more accessible, albeit less comprehensive, tool for clinicians and researchers. Future versions could potentially incorporate 3D analysis techniques to offer a more holistic assessment.

S-BEST is a new photometric analysis program that automatically localizes landmarks and measures them in a few seconds on modern computers. Our study has shown that S-BEST is accurate for widely used photometric measurements and breast asymmetry indices. S-BEST can be a useful tool for both clinical and research purposes, providing valuable information for patient counseling, surgical planning, and outcome evaluation. Further studies are needed to validate its accuracy and investigate its applicability to other breast conditions.
